# A single variant sequencing method for sensitive and quantitative detection of HIV-1 minority variants

**DOI:** 10.1038/s41598-020-65085-y

**Published:** 2020-05-18

**Authors:** Gurjit Sidhu, Layla Schuster, Lin Liu, Ryan Tamashiro, Eric Li, Taimour Langaee, Richard Wagner, Gary P. Wang

**Affiliations:** 1 Division of Infectious Disease and Global Medicine, Department of Medicine, University of Florida, Gainesville, FL USA; 2 Medosome Biosciences, Alachua, FL USA; 3 Department of Pharmacotherapy and Translational Research, Center for Pharmacogenomics and Precision Medicine, College of Pharmacy, University of Florida, Gainesville, FL USA; 4 Infectious Diseases Section, Medical Service, North Florida/South Georgia Veterans Health System, Gainesville, FL USA; 5 Present Address: Department of Medicine, St. Luke’s Hospital, Chesterfield, MO USA

**Keywords:** Infectious-disease diagnostics, HIV infections

## Abstract

HIV drug resistance is a major threat to achieving long-term viral suppression in HIV-positive individuals. Drug resistant HIV variants, including minority variants, can compromise response to antiretroviral therapy. Many studies have investigated the clinical relevance of drug resistant minority variants, but the level at which minority variants become clinically relevant remains unclear. A combination of Primer-ID and deep sequencing is a promising approach that may quantify minority variants more accurately compared to standard deep sequencing. However, most studies that used the Primer-ID method have analyzed clinical samples directly. Thus, its sensitivity and quantitative accuracy have not been adequately validated using known controls. Here, we constructed defined proportions of artificial RNA and virus quasispecies and measured their relative proportions using the Primer-ID based, quantitative single-variant sequencing (qSVS) assay. Our results showed that minority variants present at 1% of quasispecies were detected reproducibly with minimal variations between technical replicates. In addition, the measured frequencies were comparable to the expected frequencies. These data validate the accuracy and reproducibility of the qSVS assay in quantifying authentic HIV minority variants, and support the use of this approach to examine the impacts of minority HIV variants on virologic response and clinical outcome.

## Introduction

There are approximately 37.9 million people living with HIV^1,2^. As antiretroviral drug coverage increases and patients become more treatment experienced, the prevalence of HIV drug resistance is expected to increase^3^. The presence of drug resistant HIV in the viral population is known to compromise virologic response to ART^4–6^. Currently, genotypic and phenotypic assays are used to identify HIV drug resistance in clinical settings. However, because phenotypic assays are time-consuming and expensive, they are generally reserved for patients with known or suspected complex drug-resistance patterns. In contrast, genotypic testing by Sanger Sequencing is the preferred approach for most patients beginning or switching ART. While the Sanger Sequencing method is informative in many settings, it is insensitive in detecting minority variants circulating in less than 20% of the viral quasispecies^7,8^. Many studies have shown that minority drug resistant variants can compromise response to therapy^5,6,9–22^. However, the level at which minority variants become clinically relevant remains unclear.

Numerous studies have used deep sequencing methods such as Roche/454 pyrosequencing and Illumina sequencing to detect minority HIV variants^23–30^. However, even with the next generation sequencing (NGS) technology, the original quasispecies structure may be distorted by technical artifacts during sample preparation, which may incorporate errors during reverse transcription and PCR, resampling errors from low copy number of viral templates, PCR amplification bias, and sequencing errors from Roche/454 or Illumina sequencing. Thus, quantification of minority variants using standard deep sequencing approaches may not be accurate.

Several years ago, Jabara *et al.*^31^ developed a Primer-ID approach which solved many of the technical artifacts and biases described above. This approach relies on the use of random sequence tags in the reverse transcription primer such that each RNA template from the original quasispecies population receives a unique Primer-ID. After sequencing, datasets are clustered according to the unique Primer-ID, and a consensus sequence for each template is generated. Since each starting RNA template is tagged with a unique Primer-ID barcode, errors originating from subsequent steps such as nucleotide misincorporation and deep sequencing, allelic skewing from differential amplification, and template resampling, are corrected using this approach. Thus, the resulting population of consensus sequences should accurately represent the original quasispecies population, and the approach holds great promise for quantifying minority variants in clinical samples. However, most published studies have applied the Primer-ID method directly to clinical samples^27,32–37^. The sensitivity, reproducibility, and quantitative accuracy of the approach have not been adequately validated using quasispecies of known proportions.

In the present study, we constructed artificial RNA pools and artificial viral quasispecies and determined the frequency of minority HIV variants using a Primer-ID, quantitative single variant sequencing assay. The goal was to validate using known controls that the assay can detect minority variants reproducibly at or above 1% of the HIV-1 quasispecies with high precision.

## Results

### Determination of the background error rate

The single variant sequencing (SVS) method leveraged the Primer-ID approach and Illumina sequencing to quantify the abundance of different variants in viral quasispecies. We first determined the background error rate for the SVS method by selecting three plasmids containing the entire HIV-1 PR gene and three plasmids containing partial RT gene segment (codon 1- 237) and subjecting each individual plasmid to *in vitro* transcription, PCR amplification, and deep sequencing using the SVS procedure (Fig. 2). We then selected three cell culture-derived HIV and subjected each virus stock to the same SVS analysis. SVS analysis was performed for the PR and RT genes for plasmids and the PR gene for viruses. Amino acids called erroneously were identified and their frequency determined at each position (see Supplemental Tables ST1 and ST2). Mean of error frequencies at each position of the given sequence was calculated and reported as the background error rate per position for that particular gene. For both plasmid-derived RNA and viral RNA, the background error rate per position was found to be less than 0.1% in all samples (Fig. 2).

**Figure 1:**
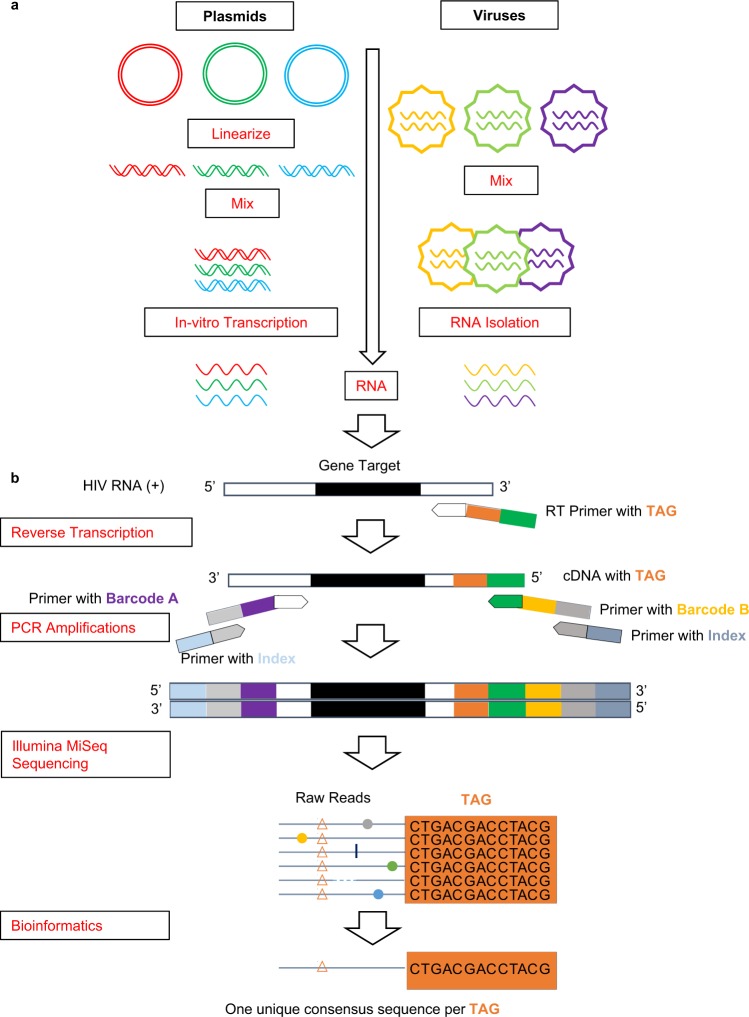
(**a**) Generation of artificial pools of RNA quasispecies or virus quasispecies. (left) Plasmids encoding PR or RT gene segments were linearized, then mixed in different proportions to generate artificial pools. RNA quasispecies was generated by *in vitro* transcription. (right) Viral particles were mixed in different proportions to generate virus quasispecies, then viral RNA was extracted. **(b)** The frequency of each RNA quasispecies was quantified using single variant sequencing, leveraging the primer-ID approach and high-throughput Illumina sequencing.

**Figure 2:**
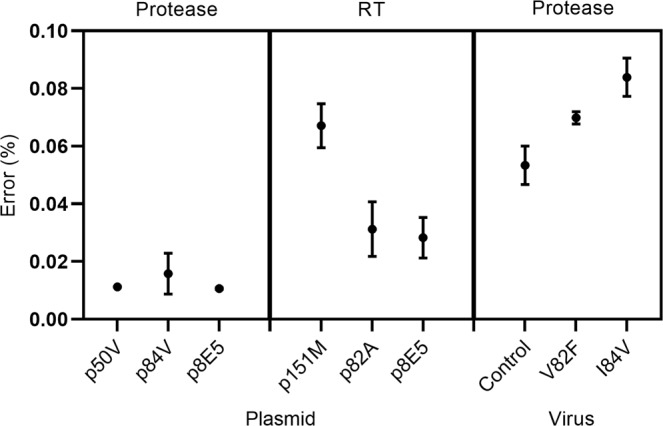
Background error rate of the single variant sequencing (SVS) method. Purified plasmids (in triplicate) or viruses (in duplicate) were subjected to the SVS analysis. For each of the three plasmids, RNA encoding HIV-1 PR (amino acids 8–99) or RT (amino acids 11–133) was generated by *in vitro* transcription, followed by the SVS procedure and Illumina sequencing. For each of the three virus stocks, viral RNA was extracted, then subjected to SVS and Illumina sequencing of the PR gene segment (amino acids 8–99). Frequency of amino acids called erroneously at all sequenced positions was scored and plotted as % mean error per position for the sequenced gene. Mean with SEM is shown.

### Sensitive and quantitative detection of individual variants in plasmid-derived RNA quasispecies

To determine the threshold at which individual minority variants could be detected consistently, we generated artificial pools of RNA quasispecies derived from plasmids of known sequences. The concentration of each linearized plasmid was determined, and combined in different ratios to generate 3 plasmid populations with defined proportions (Supplemental Table ST4). Two of the three populations included minority species at 1% mean abundance (i.e. pools B and C for PR; pools E and F for RT). RNA quasispecies was then generated by *in vitro* transcription of the plasmid pools. To evaluate the contribution of pipetting variations during the construction of artificial pools, each pool was prepared in quadruplicates by manual pipetting and also by a liquid handling robot.

**Figure 3:**
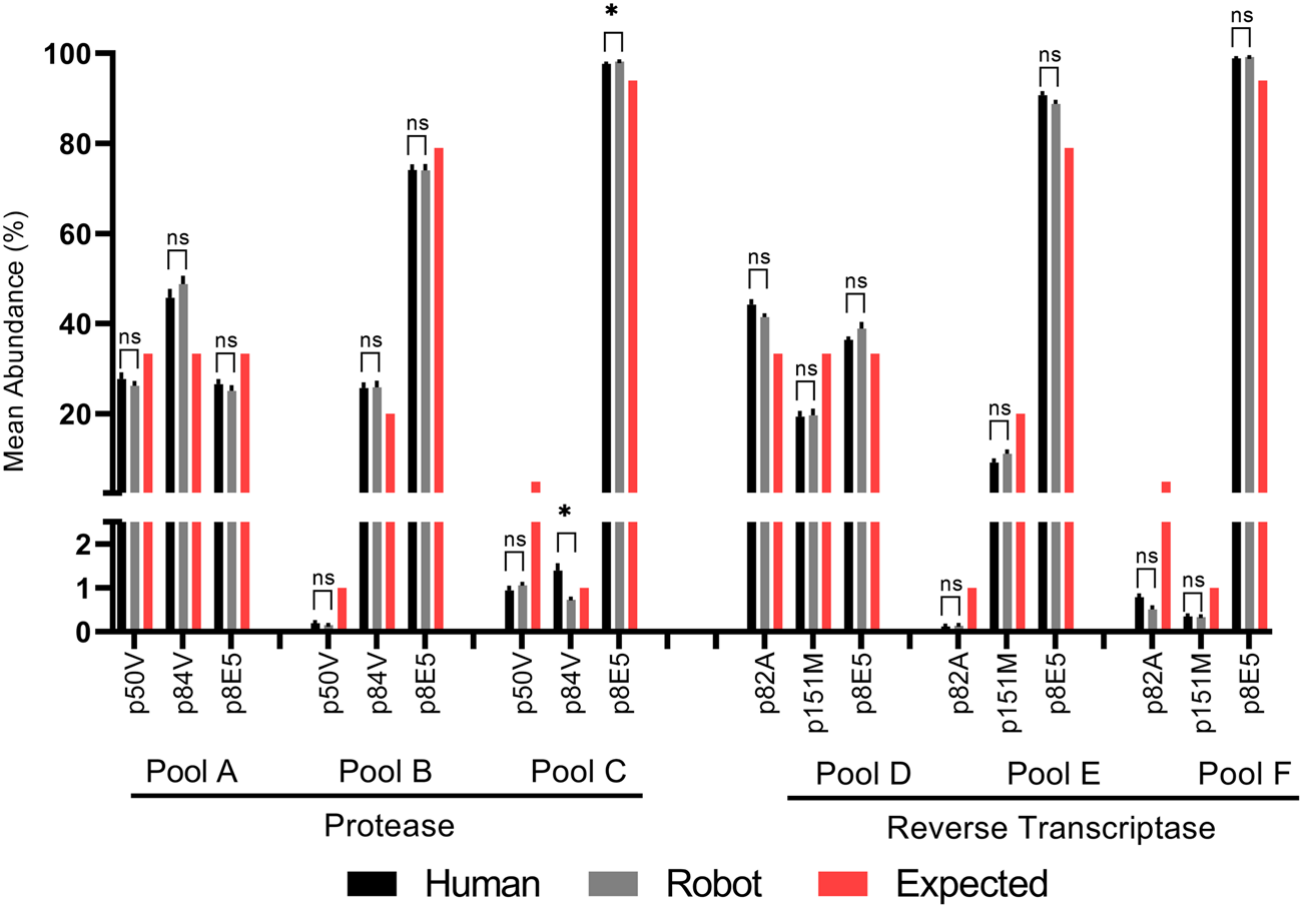
Frequency of individual variants in quasispecies pools determined using the SVS procedure. Each artificial RNA quasispecies is shown as a panel (pools **A–F**). Each bar within the panel represents the abundance of an individual variant, and the red bar denotes the expected frequency calculated based on the initial plasmid concentration. Each plasmid pool was prepared in quadruplicates using a liquid handling robot (gray) or manual pipetting (black). Individual variants in the PR (left) or RT (right) genes were analyzed and the mean abundance (%) with SEM of each variant was plotted. ns = not statistically significant. *= p < 0.005.

The abundance of individual variants in each PR or RT artificial RNA pool is shown in Fig. 3. The expected (theoretical) frequency of individual variants in each pool was calculated based on measured concentrations of linearized plasmids. The SVS analysis revealed that minority variants present at mean 1% abundance of the quasispecies populations (i.e. Protease pool B p50V, Protease pool C p84V, Reverse Transcriptase pool E p82A, and Reverse Transcriptase Pool C p84V) were detected in all replicates, although the measured mean abundance deviated from the expected abundance in some cases (observed mean abundance - Protease pool B p50V: 0.14%, Protease pool C p84V: 1.70%, Reverse Transcriptase pool E p82A: 0.08%, and Reverse Transcriptase Pool F p151M: 0.33%). We observed minimal differences between technical replicates, and also between the two pipetting methods (liquid handling robot vs manual pipetting). These results indicate that the SVS method reproducibly detects minority variants present at or above 1% abundance of quasispecies.

### Sensitive and quantitative detection of individual variants in virus-derived RNA quasispecies

**Figure 4:**
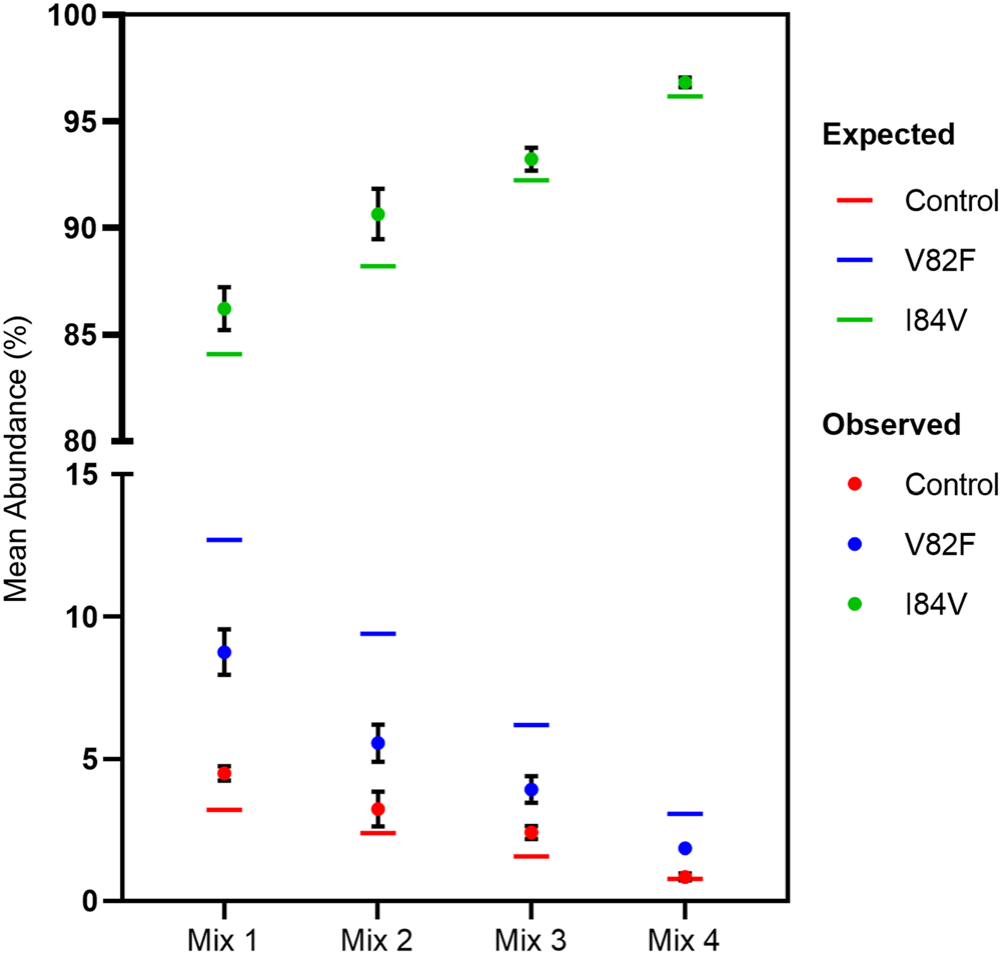
Frequency of individual variants in virus quasispecies determined using SVS. Mean abundance (%) of each variant in the artificial viral quasispecies (mix 1–4) is shown on the y-axis. The horizontal line denotes the expected frequency. Each virus pool was prepared in quadruplicates and amplified and sequenced independently. Mean with SEM is shown.

To demonstrate that the SVS method can be applied to viral populations, we selected three cell culture-derived viruses that differed in two positions (82 and 84) in the PR gene and constructed four artificial viral mixtures with defined proportions (Supplemental Table ST5). Each artificial pool consists of two minority variants ranging from 0.8–12.7% and one majority variant (>84.1%). The abundance of each virus was determined using SVS and compared with the expected (theoretical) abundance calculated based on p24 titers. The analysis showed that minority variants (i.e. control virus) present at 1 to 3% of the expected frequency were detected reproducibly and were comparable to the expected frequencies (4.49% vs 3.21% expected, 3.23% vs 2.38% expected, 2.41% vs 1.57% expected, 0.84% vs 0.77% expected; mix1 to mix4, respectively) (Fig. 4). In addition, variations in measured frequencies between technical replicates were small. These results demonstrate that the SVS procedure reproducibly detected minority variants at or above ~1% of the viral quasispecies.

**Figure 5:**
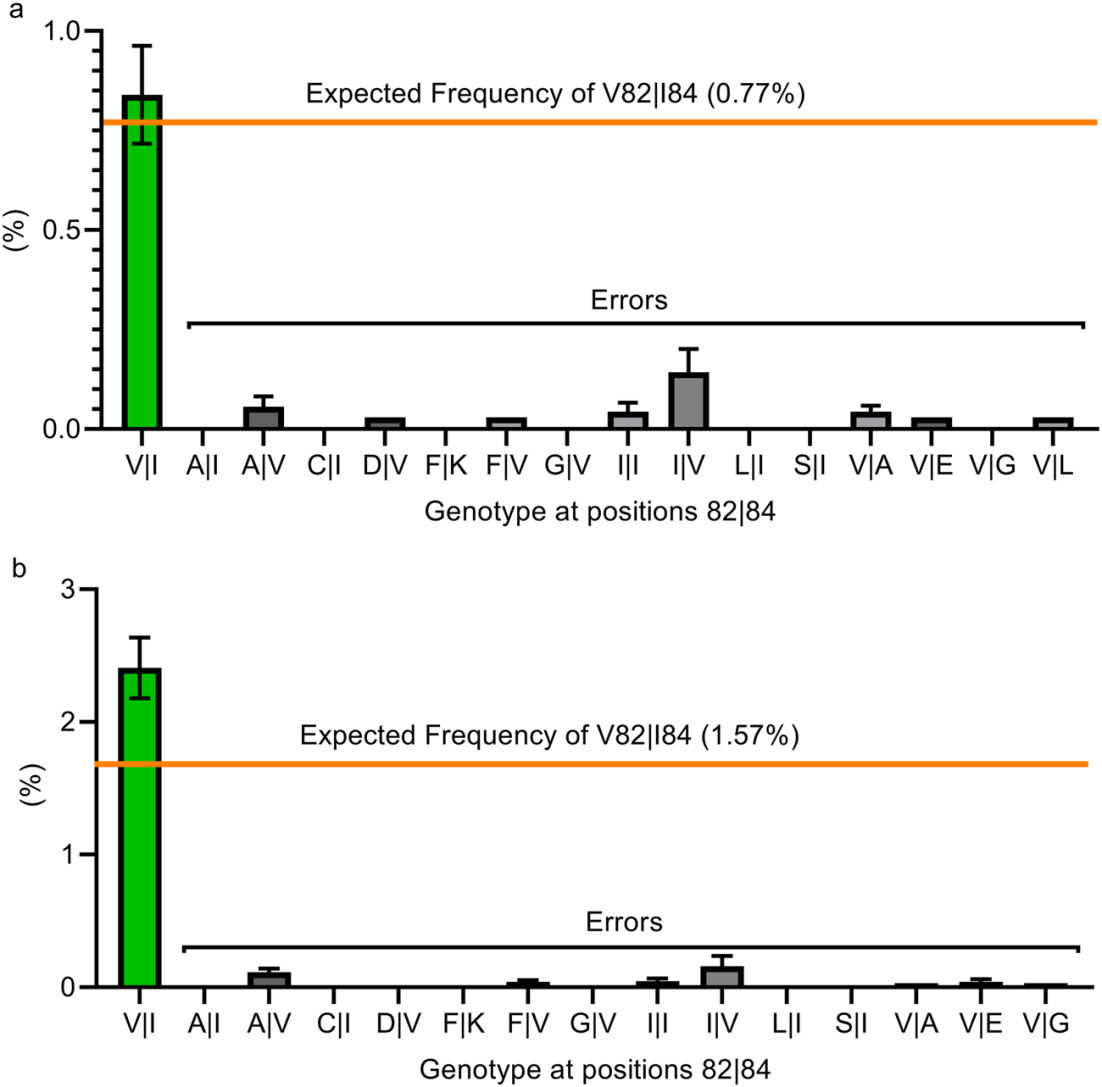
Detection of low frequency authentic minority variants in viral quasispecies. (**a**) Observed mean abundance (%) (green bar) vs. expected frequency (orange line) of Control (V82 | I84) virus in Mix 4. (**b**) Observed mean abundance (%) (green bar) vs. expected frequency (orange line) of Control (V82 | I84) virus in Mix 3. Gray bars represent the frequency of viruses with amino acids called incorrectly at positions 82 and 84. Each sample was performed in quadruplicate. Mean with SEM was plotted.

To further confirm the high sensitivity and accuracy of the SVS assay, the frequencies of authentic minority variants in virus Mix 4 and Mix 3 were compared to the frequencies of reads that were called erroneously (Fig. 5). The frequency of authentic minority variants at position 82 and 84 called correctly (green bars; 0.77% and 2.41% in virus Mix 4 and Mix 3, respectively) was significantly higher than the frequencies of erroneous calls (variant with the highest frequency has a mean abundance of 0.143% for Mix 4 and 0.158% for Mix 3; p < 0.00001). Overall, >99.6% of the reads were called correctly in all four mixes. These results indicate that authentic minority variants present in as low as 0.8% frequency in the viral quasispecies could be distinguished accurately from the background errors.

## Discussion

HIV-1 drug resistant minority variants can compromise response to antiretroviral therapy. Although many studies have investigated the clinical impact of minority variants, the clinical significance of HIV-1 drug resistant minority variants continues to be debated^27,29,30,37–40^. Furthermore, the level at which drug resistant minority variants become clinically relevant remains unclear. The Primer-ID method is one approach that can reduce accumulation of erroneous variants^41^. Correctly calling minority variants while simultaneously reducing erroneous base calls is paramount for accurate determination of minority drug-resistance mutations and studies of their impacts on treatment response. Here, we leveraged the Primer-ID approach and Illumina deep sequencing and report the validation of a quantitative Single Variant Sequencing (SVS) method for accurate and sensitive detection of minority HIV-1 variants. We constructed artificial RNA quasispecies of defined proportions by *in vitro* transcription of plasmids and direct extraction of cell culture-derived viruses, then interrogated the proportions of RNA quasispecies using the SVS methods.

To accurately identify and quantify authentic HIV-1 minority variants, it is critical that an assay can reliably distinguish authentic low frequency variants from errors generated during nucleic acid amplification and the deep sequencing process. We first mixed plasmids carrying HIV-1 PR and RT gene segments with known mutations, then generated RNA quasispecies by *in vitro* transcription of the plasmid pools. Expected proportions of different plasmids were calculated based on plasmid concentrations to include one or two minority variants (less than 20%) and one or two majority variants (20% or higher). Our results showed that the assay was highly sensitive in accurately detecting minority variants in as low as 1% of the quasispecies population, with a low background error rate of <0.1%. The low error rate was consistent with a recent report by Howison *et al*. that also used the Primer ID method^41^. Importantly, our data demonstrated a high level of reproducibility among technical replicates.

In some cases, the abundance of variants (both majority and minority variants) deviated from the expected abundance calculated based on measured plasmid concentrations. The deviations for individual quasispecies were consistent among technical replicates in all quasispecies (i.e. reproducibly higher or lower than the expected values and with a similar magnitude) (Fig. 3). We speculate that the observed differences were a result of concentration measurements of linearized plasmids, and/or stochastic events from *in-vitro* transcription (i.e. differences in the amount of RNA transcribed *in vitro* from different plasmids). Seifert *et al*.^42^ combined Primer ID with MiSeq sequencing to study heterogeneous HIV-1 populations by mixing five viruses in equal proportions (20% each) calculated based on RNA copy numbers. However, they observed proportions ranging between 6% and 38%. Similar deviations were reported by Howison *et al*^41^ in their analysis of artificial quasispecies based on RT-PCR or plasmid DNA measurements. Taken together, these data suggest that *in vitro* plasmid or RNA-based quasispecies may be adequate for the evaluation of assay sensitivity, but may not be sufficiently robust for determining the quantitative accuracy of variant populations in quasispecies. Going forward, an improved method for constructing accurate proportions of viral quasispecies is essential for validating the quantitative accuracy of the SVS assay.

To demonstrate that the SVS method is also applicable to viral populations, we generated defined mixtures of HIV-1 virus quasispecies. To minimize the impact of concentration measurements on accurate proportions of each viral population, we first combined two variants at 1:4 ratio, and then spiked the mixture into the third virus variant to generate four viral quasispecies consisting of two minority variants in a fixed ratio (1:4) ranging from 0.8% to 12.7% abundance and one majority variant at >84% abundance. This approach was expected to generate different quasispecies with a fixed ratio between the two minority variants. Indeed, our data (Fig. 4) demonstrated a consistent ratio between the two minority variants across all four viral quasispecies, with the observed ratio (1:2) deviating slightly from the expected value (1:4). This high level of consistency across the four quasispecies suggests that the differences likely resulted from an over or under estimation of viral RNA copy number based on p24 measurements. Overall, the SVS assay demonstrated high reproducibility in identifying and quantifying both majority and minority variants across different sample types with minor errors that do not impact data interpretation. Importantly, these results demonstrate that the SVS method can consistently quantify authentic minority variants present in as low as 0.8% of the viral quasispecies.

This study has several limitations. First, the initial Primer-IDs label individual RNA templates during reverse transcription. The SuperScript IV Reverse Transcriptase possesses no proofreading activity and may have introduced errors during the first strand cDNA synthesis which could not be corrected in subsequent steps^43^. Second, PCR amplification following cDNA synthesis may have introduced additional errors in the Primer-ID barcode sequence, thereby generating offspring Primer-IDs, the effect of which could be minimized using a cutoff model proposed by Zhou *et al*^44^. However, the use of high-fidelity Platinum SuperFi DNA Polymerase should reduce the likelihood of additional errors introduced during the PCR step.

In summary, we have validated the SVS method using known controls, and showed that the assay consistently detects minority variants at or above 1% level with high precision. These results support the use of quantitative single variant sequencing assay to examine the impacts of minority HIV-1 drug resistant variants on virologic response and clinical outcome.

## Methods

### Construction of artificial RNA pools with defined proportions

Plasmids (p8E5, p50V, p84V, p82A and p151M) with unique amino acid polymorphism (Supplemental Table ST7) were obtained from NIH AIDS Research and Reference Reagent Program. These constructs carry a 1060-bp fragment spanning from *gag* gene to codon 237 of the reverse transcriptase gene (nucleotide 2201 through 3261) of the HIV-1 genome (GenBank accession no. K03455) in pCR 2.1-TOPO vector (Invitrogen). To confirm the sequence of the insert, plasmids were transformed into *E. coli* (One Shot TOP10 Competent Cells, ThermoFisher, Cat # C404010), single colonies were selected and grown in Luria Broth with 25 μg/ml kanamycin, and plasmid DNA was isolated using NucleoSpin Plasmid (NoLid) kit (Macherey-Nage, Cat # 740499). The sequence of the PR and RT gene segments in each plasmid was confirmed by Sanger sequencing using following primers; PRC (forward): 5’-CTCCCCCTCAGAAGCAGGAGCCGATAGACAAGGAACTGTATCC and RT3 (reverse): 5’-TATCAGGATGGAGTTCATAAC. Next, plasmids were linearized using *Bam*HI-HF restriction enzyme (New England Biolabs), and the length of the linearized plasmid was verified by agarose gel electrophoresis and DNA concentration measured using the Qubit dsDNA HS Assay Kit in Qubit 4 fluorometer (Invitrogen). Purified linearized plasmids were mixed to create 12 pools with defined proportions based on measured DNA concentrations (Supplemental Table ST4; Fig. 1a). Six pools were prepared by hand pipetting, and six identical pools were constructed by robot pipetting using Eppendorf epMotion 5070 liquid handling robot.

To generate artificial RNA pools with defined proportions (Supplemental Table ST4; Fig. 1a), individual plasmids or plasmid pools were transcribed *in vitro* using T7 RiboMAX Express Large Scale RNA Production System (Promega, Cat # P1320), and the transcribed RNA was purified using the NucleoSpin RNA Clean-up Kit (Macherey-Nagel, Cat # 740948). The RNA concentration was measured using the Nanodrop 2000 (Thermo Fisher) and adjusted to 100,000 copies/μL for subsequent reverse transcription and PCR.

### Construction of virus pools of defined proportions

HIV-1_RF_ (Cat# 2806; Control; V82, I84), HIV-1_RF/L323-12-3_ (Cat# 2806; V82F) and HIV-1_RF/L323-9-1_ (Cat# 2807; I84V) (cell-free culture supernatant) were obtained from the NIH AIDS Reagent Program, Division of AIDS, NIAID, NIH^45,46^, contributed by Dr. Dean Winslow. To construct the virus pools with defined proportions, the p24 content was determined using the HIV1 p24 ELISA Kit (Abcam, Cat # ab218268), then 10 μL each from Control (#2803) and V82F (#2806) were combined to obtain a mixture with 1 to 3.96 ratio (Control and V82F) based on p24, then this mixture was added in differing ratios to I84F (#2807) to generate four virus pools with known proportions (Supplemental Table ST5; Fig. 1a). Viral RNA was isolated using the QIAamp Viral RNA Mini Kit (Qiagen, Cat # 52904).

### Single variant sequencing (SVS)

A schematic of the SVS procedure is shown in Fig. 1b, which included reverse transcription, PCR amplification, pooling, and Illumina sequencing. The SVS procedure utilizes a unique design of reverse transcription (RT) primers for priming the first strand of cDNA synthesis. Each RT primer molecule includes a 14 random nucleotide sequence constituting a unique Primer-ID Tag, which is flanked by a sequence at the 3’ end that anneals to the RNA template and a sequence at the 5’ end that serves as the annealing site for PCR primers (Supplemental Table ST6). The RNA from *in vitro* transcription or RNA isolated from virus particles (Fig. 1a) serves as the template for cDNA synthesis using SuperScript IV Reverse Transcriptase (SuperScript IV First-Strand Synthesis System, Invitrogen) and amplicon (protease or reverse transcriptase) specific RT primers that contained the Primer-ID tag. Each reaction contained 10^2^-10^3^ fold molar excess of primers to ensure that each RNA template was reverse transcribed to generate cDNA labeled with a unique primer ID tag. The resulting cDNA was purified using NucleoSpin Gel and PCR Clean-up (Macherey-Nagel, Cat# 740609), then amplified using Platinum SuperFi DNA polymerase (Invitrogen) with forward and reverse PCR primers that included 4–8 variable length nucleotide barcode sequences specific to each sample. The PCR products were separated by agarose gel electrophoresis, purified using NucleoSpin Gel and PCR Clean-up (Macherey-Nagel, Cat# 740609), and tailed with index sequences required for Illumina sequencing. Amplified DNA were combined in equimolar pool, gel purified and quantified by qPCR (KAPA Library Quantification kit for Illumina sequencing platforms, Kapa Biosystems), then sequenced on the Illumina MiSeq using the v3 600 cycle kit and a 20% PhiX spike-in. Protease (PR) gene segment (amino acids 8–99) and reverse transcriptase (RT) gene segment (amino acids 11–133) were sequenced. SVS analysis was performed for the PR and RT genes for plasmids and the PR gene for viruses.

### Bioinformatics

Raw Illumina MiSeq. 301 bp x 2 reads were de-multiplexed into individual samples according to the unique variable length barcode combination (4bp-8bp in length) on each end. Additional filtering criteria included an exact match to PCR primer sequences, an average quality score of 30 or higher (<0.001 error rate in raw reads), and a minimum length of 270 bp for each paired-end read. Each paired-end read was joined using FLASh (http://ccb.jhu.edu/software/FLASH/) with a minimum of 10 base overlap. For each sample, the joined reads were grouped by the unique 19-bp tags (including 5 bp control bases) introduced during reverse transcription. Consensus sequences were called for each unique tag via alignment using MAFFT (http://mafft.cbrc.jp/alignment/software/) when three or more reads share the same tag. Consensus sequences that contained ties at certain positions resulting in degenerate bases were excluded due to ambiguity. The resulting consensus sequences were aligned against corresponding reference sequences and manually inspected and corrected for artificial gaps introduced from the sequencing process. Translation of codons and summarization of mutations were carried out using custom scripts in R (https://www.r-project.org/) with the BioStrings package (http://bioconductor.org/packages/release/bioc/html/Biostrings.html).

## Supplementary information

Supplementary information.

Supplementary information1.

Supplementary information2.

Supplementary information3.
