# Supplementary material for: A single variant sequencing method for sensitive and quantitative detection of HIV-1 minority variants
Source: Sci Rep. 2020 May 18;10:8185. doi: 10.1038/s41598-020-65085-y (PMC7234988; doi:10.1038/s41598-020-65085-y)
Supplement: Supplementary file 1 — Supplementary information. [file 41598_2020_65085_MOESM1_ESM.pdf]

A single variant sequencing method for sensitive and quantitative detection of HIV-1 minority variants

Gurjit Sidhu[1,#], Layla Shuster[1,2,#], Lin Liu[1,^], Ryan Tamashiro[1], Eric Li[1], Taimour Langaee[3], Richard Wagner[2], Gary P Wang[1,4,*]

Supplemental Table ST4: Theoretical or expected abundance (%) of *in vitro* transcribed RNA encoding protease or reverse transcriptase in artificial RNA pools, calculated based on plasmid concentration

**Plasmid**	**Protease**			**Plasmid**	**Reverse Transcriptase**		
	Pool A	Pool B	Pool C		Pool D	Pool E	Pool F
p50V	33.3	1.0	5.0	p82A	33.3	1.0	5.0
p84V	33.3	20.0	1.0	p151M	33.3	20.0	1.0
p8E5	33.3	79.0	94.0	p8E5	33.3	79.0	94.0

Supplemental Table ST5: Theoretical or expected abundance (%) of HIV variants in artificial mixtures of cell culture-derived HIV, calculated based on p24.

Pool	Control	V82F	I84V
Mix 01	3.2	12.7	84.1
Mix 02	2.4	9.4	88.2
Mix 03	1.6	6.2	92.2
Mix 04	0.8	3.1	96.2

Supplemental Table ST6. Primers used in this study

Primer	Sequence 5'-3'	Comment
HIV1-BC-PI-ID-RT	TGACTCACGAGTCATCGACTGCAGGCAGAT**NNNNNNNNNNNNNN**VVVVBGGTACAGTTTCAATRGGACT	Protease cDNA primer with **Primer-ID Tag**
HIV1-BC-RT1-ID-RT	TGACTCACGAGTCATCGACTGCAGGCAGAT**NNNNNNNNNNNNNN**VVBVBCTAGGTATGGTRAATGCAGT	Reverse Transcriptase cDNA primer with **Primer-ID Tag**
HIV1-BC-PI-IF	CTACACGACGCTCTTCCGATCT**-Barcode-**TCCCTCARATCACTCTTTGGCA	1st round PCR Forward Primer with **Barcode** for Protease amplicon
HIV1-BC-RT1-IF	CTACACGACGCTCTTCCGATCT**-Barcode-**TCCYATTGARACTGTACCAGT	1st round PCR Forward Primer with **Barcode** for Reverse Transcriptase amplicon
HCV-ID-IR	TGCTGAACCGCTCTTCCGATCT**-Barcode-**CATCGACTGCAGGCAGAT	1sr round PCR Reverse Primer with **Barcode**
PE1	AATGATACGGCGACCACCGAGATCTACACTCTTTCCCTACACGACGCTCTTCCGATCT	2nd round PCR forward primer with illumina adapter and index

PE2	CAAGCAGAAGACGGCATACGAGATCGGTCTCGGCATTCCTG CTGAACCGCTCTTCCGATCT	2nd round PCR reverse primer with illumina adapter and index

Supplemental Table ST7. Amino acid polymorphisms in PR and RT gene segments in plasmids used in this study

Protease Amplicon																				
	10	14	20	35	36	37	41	46	50	54	62	63	71	73	77	82	84	85	90	93
p8E5	L	K	K	E	M	S	R	M	I	I	I	L	A	G	V	V	I	I	L	I
p50V	I		T	D	I			I	V	M	M	P	V			I		V		
p84V	I	R				N	K					P	V	S	I		V		M	L
RT Amplicon																				
	20	67	68	70	77	90	116	118												
p8E5	R	D	S	K	F	V	F	V												
p82A	K	N		R		I														
p151M	K		G		L		Y	I												
